# The Application of Flipped Classroom Strategies in Medical Education: A Review and Recommendations

**DOI:** 10.1007/s40670-024-02166-x

**Published:** 2024-10-03

**Authors:** Priti L. Mishall, Eiman M. Abdel Meguid, Ihsan A. Elkhider, Mohammed K. Khalil

**Affiliations:** 1 Department of Pathology, Albert Einstein College of Medicine, Bronx, NY 10461 USA; 2 Department of Ophthalmology and Visual Sciences, Albert Einstein College of Medicine, 1300 Morris Park Ave, F620SC, Bronx, NY 10461 USA; 3 Centre for Biomedical Sciences Education, School of Medicine, Queen’s University Belfast, Dentistry & Biomedical Sciences, Belfast, UK; 4 University of South Carolina School of Medicine Greenville, Greenville, SC 29605 USA; 5 Department of Biomedical Sciences, University of South Carolina School of Medicine Greenville, Greenville, SC 29605 USA

**Keywords:** Critical thinking, Active learning, Blended learning, Flipped classroom, Medical education

## Abstract

The role of a flipped classroom (FC) instructional method has been expanding in medical education. Despite recent interest in FC pedagogy, little is known about optimal FC implementation strategies in medical education and their impact on learning outcomes. This article aimed to outline the educational theories that guide the design of the flipped instruction method and review the relevant literature to provide evidence-based recommendations for planning, designing, developing, implementing, and evaluating FC instruction in medical education. The review incorporates evidence-based literature that highlights students’ performance outcomes and perceptions of the FC instruction method in medical education.

## Introduction

The recently emerged flipped classroom (FC) pedagogy increases students’ engagement with instructional materials. In the FC approach, students can practice lower levels of Bloom’s taxonomy through pre-class material and faculty can design in-class time on higher level Bloom’s taxonomy [1], thus increasing the amount of active [2, 3] and deep learning [4, 5]. Additionally, the FC instructional method represents a shift from teacher-centered to learner-centered instructional strategies [6].

The FC learning process consists of both out-of-class and in-class activities. The out-of-class activities are pre-class assignments guided by static or technology-enhanced instructional materials. The in-class activities consist of non-digital [7, 8] or digital applications [9, 10] of the instructional content: the non-digital activities include collaborative team-based, case-based, or discussion-based activities, and the digital activities include innovative technologies, such as audience response systems, Kahoot, and game-based learning platforms [11].

Although this basic design of FCs is consistent, there is variability in how instructors implement the FC model, specifically in the time allocated for out-of-class activities, the expectations for in-class activities, and the style of assessing performance through formative and summative examinations. The specific approach used in a course may vary depending on the context, but FC pedagogy requires that instructors provide quality out-of-class materials that facilitate the learning process so students can achieve a better understanding of the content. These materials should support the students’ abilities to pace themselves, engage with the content, manipulate models, select tasks, and reflect and respond to questions and prompts [12, 13].

There are numerous possible implementations of the FC in teaching and learning; however, most existing studies focused on courses relevant to the STEM (science, technology, engineering, and mathematics) disciplines [14, 15]. Though the role of FC in medical education has grown steadily over the last decade [16], very few evidence-based FC models exist for anatomy [17, 18], histology [19], physiology [20], embryology [21], neuroanatomy [22], and medicine [23, 24]. Furthermore, despite the recent interest in FC instruction, little is known about the optimal FC implementation strategies and the impact of the model on learning outcomes; hence, evidence on the effectiveness of the FC model remains limited [19]. The goals of this narrative review article were to define FC, discuss FC-related educational theories and instructional design principles, and highlight recommendations for effective FC implementation in medical education. Furthermore, the article covers the gaps or controversies in implementing flipped classrooms. Although several studies [17, 25] demonstrate students’ positive perceptions of FC experience, most of these studies measure student perception using Likert scale but do not inform the impact of FC on student performance. Some studies use pre-test and post-test which are low-value ways to assess student retention of content [26]. Knowledge of these limitations will inform the readers on innovative ways to design medical education research.

## Definition of Flipped Classroom

FC instruction is a pedagogical approach that directs instruction to the individual learning space and transforms it into a dynamic learning environment, replacing traditional teacher-centered teaching with student-centered learning. It makes students responsible for their own learning and stimulates active engagement in in-class activities and online self-learning [27]. The FC facilitates interaction between instructors and students and promotes differentiated learning as it includes not only videos, lectures, and homework but also problem-, game-, and task-based learning activities [28–30].

There are substantial variations in the definitions of FC in the literature. However, the aims of the different FC approaches are almost identical: e.g., freeing up class time; engaging students in effective, autonomous, and interactive learning; enhancing students’ learning performance; and fostering high-order thinking capabilities [27, 31]. Lage et al. [30] provided a clear definition of FC: “Inverting the classroom means that events that have traditionally taken place inside the classroom now take place outside the classroom and vice versa.” Later, Bergmann and Sams [28] noted that terms such as “blended learning,” “reverse instruction,” “inverted classroom,” and “24/7 classroom” are interchangeably used to refer to FC-based instruction.

Broadly, the FC is an instructional method in which learners independently acquire foundational knowledge outside the classroom through self-directed learning. Learners then use this foundational knowledge during in-class activities to analyze, apply, and solve complex course content through peer interactions under the instructor’s guidance [14, 25]. Abeysekera and Dawson [7] distilled the core characteristics of FC down to (a) studying foundational knowledge via self-directed pre-class activities supported by technology (e.g., video) and (b) in-class activities that are devoted to more complex contents and involve group interactions and instructor guidance.

The simplest implementation of FC is to replace after-class homework with the expectation that students study learning materials prior to class. Moreover, the out-of-class activities are often supplemented or integrated with instructional videos [32].

## Theoretical Foundation of Flipped Classroom Strategies

A wide range of educational theories provides guiding principles for the design and implementation of the FC instructional method, specifically for effective in- and out-of-class activities. However, the FC instructional method is mainly guided by two relevant educational theories, namely adult learning theory or andragogy [33], and social constructivist theory [34].

### Adult Learning Theory

The following assumptions of adult learning theory or andragogy [33, 35] provide and explain the major learning principles utilized in FC learning experiences: adult learners (1) are internally motivated to learn, (2) prefer self-directed learning approaches, and (3) are problem-centered and interested in the application of the knowledge, (4) adult learners’ prior experiences assist them with learning, and (5) adult learners exhibit a readiness to learn tasks oriented to their social roles [33].

#### Adult Learners are Internally Motivated to Learn

Learning is driven by extrinsic and intrinsic motivation. Extrinsic motivation is influenced by external factors, such as grades, prizes, and extra credit, whereas intrinsic motivation is influenced by inherent factors, such as self-esteem, curiosity, enjoyment, and relatedness to the task [36]. Research has found that FC activities increased students’ intrinsic and extrinsic motivation by improving their competence, autonomy, and ability to relate to the tasks [7]. In an FC pedagogy, students who choose to undertake and accomplish out-of-class activities will be more intrinsically motivated than those who are externally compelled to do the work [7].

#### Adult Learners Prefer Self-Directed Learning Approaches

Self-directed learning is an important skill that the learners of the twenty-first century must develop because today’s healthcare professionals are expected to identify their learning needs through their daily contact with patients and then use a variety of resources to meet these needs [37]. Moreover, Sanders and Walsh emphasize that facilitated developmental support from faculty is essential to provide a structured learning framework to the learners [38]. The FC out-of-class activities curated by the instructor provides a structured learning framework for self-directed learning activities. The FC models grant learners more control over their learning, and this independence and flexibility with learning promotes learner engagement with the material [39].

#### Adult Learners are Problem-Centered and Interested in the Application of Knowledge

Adults prefer immediate application of what they learn. The in-class problem-based and case-based activities in FC give students an opportunity to apply their learnings.

#### Adult Learners’ Prior Experiences Assist them with Learning

Adults draw a lot of meaningful learning from their past experiences including their mistakes. The FC out-of-class activities should help learners draw from their past experiences, whereas the in-class activities should be an extension of the scaffolding built into the out-of-class activities.

#### Adult Learners Exhibit a Readiness to Learn Tasks Oriented to their Social Roles

Learning activities should be in the context of the tasks performed by the learners in the real world. FC in-class activities orient learners increasingly to the developmental and social roles as a physician. Therefore, adult learners are ready to learn concepts and skills that will equip them to cope effectively with real-life situations [40].

### Social Constructivist Theory

Social constructivist theory views learning as a social process in which learners actively construct and apply knowledge as they interact with their environment [41]. Social constructivist theory applies to the in-class activities of the FC. The in-class activities are designed to involve interaction between peers, for example, a think-pair-share activity [11], cooperative learning [42], team-based learning [43], and collaborative learning [44]. These activities which involve peer interactions via discussions play a vital role in increasing learners’ abilities to test their ideas, synthesize the ideas of others, and build a deeper understanding of what they are learning [45]. The in-class activities in FCs promote collaborative learning, which promotes retention and in-depth processing associated with the cognitive manipulation of information [46]. In class, instructors serve as facilitators of learning by developing interactive experiences, challenging students to think, and providing feedback [40, 47].

## Instructional Design Principles for Flipped Classrooms

The FC model increases students’ active learning and promotes greater retention and a deeper understanding of the instructional materials [23, 48]. In a study designed to evaluate short-term knowledge retention, the residents watched online videos focused on pediatric emergencies for pre-class work and performed in-class simulation activity found that the residents performed better on post-test compared to pre-test [23]. Another study supported students’ engagement and deeper understanding of the material using FC instruction for pharmacy education. Students’ responses to the survey questionnaire revealed an increase in students’ support for learning content before class and using class time for more applied learning (*p* = 0.001). Students believed that learning key foundational content before coming to class greatly enhanced in-class learning (*p* = 0.001) [49]. In addition, the model helps learners develop critical-thinking skills by incorporating high-order Bloom’s objectives, such as analysis or evaluation [17, 50]. However, for successful FC outcomes, the planning process must follow the principles of instructional design. The following sections detail the planning, design, development, and implementation of in- and out-of-class FC activities and provide successful, evidence-based strategies. Table 1 provides a summary or checklist for FC design in medical education, and Table 2 offers a summary of the FC recommendations gathered from the reviewed literature.

**Table 1:** Checklist for designing a flipped classroom in medical education

Plan • Prepare and inform the learners of the flipped model • Prepare and chunk the content for the flipped model • Define clear course goals and learning objectives • Prepare the learning space for the flipped model
Design • Select pedagogy-based teaching strategies that promote active learning • Plan learning activities that promote deeper understanding and critical-thinking skills • Plan formative assessments to monitor the learners’ progress • Select assessment instruments to measure in-class and out-of-class learning
Develop • Use instructional design principles to develop high-quality, concise, and effective out-of-class content • Plan structured and active in-class activities that directly relate to the out-of-class content • Plan multiple opportunities to provide feedback to learners on their performance
Implement • Communicate the course objectives and expectations about grades, attendance, and assignment deadlines to the learners • Check the instructional setting to ensure learner engagement • Make sure the technology and equipment used for course delivery are available
Evaluate • Assess learning outcomes • Conduct formative and summative evaluations throughout the course • Plan to use learners’ feedback to improve the course

**Table 2:** Summary of recommendations for the flipped classroom (derived from the literature cited in the paper)

Flipped classroom activities	Recommendations
Out-of-class	1. Clearly define the learning objectives 2. Explicitly communicate the purpose of the flipped classroom 3. Communicate the link between in- and out-of-class activities to ensure that students arrive prepared for in-class activities 4. Generate self-directed, self-paced, student-centered resources: **•** Curate high-quality images from e-books, web, animations, etc **•** Organize the content in a PowerPoint and annotate or narrate the slides **•** Create high-quality videos using screen recording software **•** Add pre- and post-assignment quizzes
In-class	1. Apply active learning strategies, including case-based, team-based, and problem-based learning; roleplay involving patient–physician interactions; and questioning 2. Act as a guide to facilitate class discussions 3. Reinforce the content and engage the learners in problem-solving and critical-thinking exercises 4. Implement faculty training in developing flipped classroom pedagogy Give learners continuous feedback on their learning progress • Encourage students to learn pre-class foundational material • Hold students accountable for out-of-class activities • Grade activities online or leave them ungraded 6. Allow students to demonstrate their development of higher-order thinking skills • Assess students’ problem-solving, critical-thinking, and analytical skills • Assess their performance to move to the next stage of learning

## Initial Planning of a Flipped Classroom

Faculty who are planning to utilize FC strategies in their courses must first prepare their learners for flipped instruction, analyze the course content, and design the learning space. To prepare the learners for the flipped learning, instructors should notify the students of the new course structure and how the FC model will be implemented [51] and to incorporate student voices, consider including a student representative in the design and development of FC courses [52]. Few studies have also emphasized the importance of informing learners about the goal of a flipped course and the learning objectives for out-of-class and in-class activities [51, 53]. Although students enjoy participating in collaborative learning activities in the classroom, they tend to be less satisfied with courses lacking specific goals [54].

Successful implementation of FC requires instructors to shift the learners’ mindsets, so they understand their roles and responsibilities in the learning process [53–55]. According to Newman [54], it takes time to alter learners’ mindsets about gaining conceptual knowledge in a student-centered model instead of teacher-centered instruction. Instructors should also change their mindsets to focus on students and their learning rather than class time [56].

Learners’ extrinsic and intrinsic motivation should guide the instructor’s implementation of FC strategies. Applying the appropriate motivational strategies to stimulate, sustain, and enhance learners’ motivation to complete out-of-class activities and other tasks may considerably improve the learners’ success [52, 57].

Many studies further urge FC instructors to consider the significance of the physical space in planning their FC. The physical arrangement of the classroom space can encourage active and collaborative learning and have a great impact on learning outcomes [58–60]. Moreover, a flexible classroom design can increase spontaneous learning and facilitate different learning and teaching styles [59]. For example, a FC activity may provide each group of learners with a separate working space where they can discuss, debate, and problem-solve. Instructors can also eliminate seating rows that have learners facing the front of the classroom because the focus of the FC should be on learning, not the teacher.

## Design and Development of Instructional Materials/Activities in a Flipped Classroom

As previously established, FC involves a variety of learning resources including textbook readings, technology-mediated curated material, and self-directed out-of-class instruction followed by student-centered in-class activities for the synthesis and application of the acquired knowledge. The FC model can easily be adapted to different teaching styles, methods, situations, course objectives, and student proficiency levels [31, 61]. Ideally, the FC should aim to keep in-class contact hours and out-of-class study hours equal to the hours previously utilized in lecture-based courses [62]. Furthermore, the content and assessments of both the out-of-class and in-class activities in the FC must be aligned with clearly stated learning objectives [63, 64].

### ***Out-of-Class Activities***

The out-of-class FC activities aim to provide self-directed [33], self-paced [48, 65], and student-centered learning opportunities [47, 65]. FC instructors must establish office hours and review sessions for students, so they have the support they need. Additionally, to ensure engagement, instructors should explicitly communicate the purpose of the FC with students and make sure they are aware of the links between the out-of-class and in-class activities [66].

Out-of-class activities may include a variety of learning resources in the form of reading materials, videos, narrated PowerPoints, lecture captures, animations, or assigned reading of specific pages of a textbook [8]. Several studies have presented interesting findings on students’ preferences regarding the use of these resources. One of the studies found that students preferred annotated PowerPoint slides followed by textbook readings with guided questions and then narrated PowerPoint presentations [10]. Another study reported that 39% of students favored narrated PowerPoints [8]. It was found that the use of videos increased students’ rate of completion of out-of-class assignments compared with that of the assigned out-of-class readings [67].

Learners value out-of-class materials that are easy to access, concise, relevant, well-organized, and delivered in a variety of modalities [9, 32, 68]. One study found high-quality 20–30-min videos were particularly valued as a means of delivering content [32]. Several other studies have illustrated that students perceive videos as “easier” media to learn from compared with verbal content [48]. Moreover, videos gain maximum attention [69] and improve learning outcomes and procedural skills [70]. To engage students in the learning experience with online video modules, formative questions or quizzes help students to think more deeply about the content [24, 71]. Ultimately, high-quality out-of-class resources that enable students to self-pace and self-direct can lead to effective student engagement during in-class learning [27].

The challenge in applying the FC approach to medical education is the large amount of information that courses must cover. Chunking content and course materials, i.e., presenting smaller subsets of information with fewer components, is a practical solution that reduces cognitive load by allowing students to better manage their working memory during instruction [7, 72], so they can focus on understanding the content [73]. For additional useful strategies, van Merrienboer and Sweller [74] provided design principles that are applicable to the development and implementation of out-of-class and in-class instructional materials.

Assessments of out-of-class activities can include a range of non-graded activities, i.e., formative assessments, that inform both the students and instructor about student learning. It is important to include formative assessments when planning FC out-of-class activities [71]. Examples of formative assessments are peer feedback, student self-evaluations of their learning using a rubric or quizzes, and automated grading systems. In addition, faculty could incorporate activities that ask students to reflect on their learning progress throughout the course material. These types of practice activities help students monitor their learning and evaluate their preparedness for summative assessments. In medical education, preparing students for self-assessment of their learning can crucially contribute to their lifelong learning skills [75].

### ***In-Class Activities***

During in-class FC activities, students work together in groups to discuss and answer questions or complete activities related to the pre-class exercise. In this way, the in-class activity time can be used to reinforce and extend learning through knowledge application and problem-solving. In-class activities comprise a wide range of peer interactions and active learning methods, such as case vignettes, higher-order think-pair-shares, using audience response systems [76, 77], student group presentations, and role-playing patient–physician interactions [10] and task- and problem-based learning sessions [78–80].

The success of in-class activities depends on the experience of the instructor. Instructors can develop effective FC teaching strategies by attending faculty development workshops on FC pedagogy. During in-class activities, instructors can help students make sense of the instructional materials, respond to questions, and provide feedback on students’ learning progress. That is, instead of lecturing, the instructor acts as a guide for students by promoting their learning during in-class activities. Furthermore, studies have shown that in-class activities involving active learning techniques are effective for deep learning [5] and fostering problem-solving abilities [47, 50]. Active learning is mainly promoted in FC by improving the learners’ engagement in the learning process. FC teaching strategies in medical education that enhance learner engagement [2, 24, 33] make use of clinical cases, problem-based learning, team-based learning, and discussion activities [9, 32, 68].

Assessment of in-class activities in the FC is not limited to pop quizzes at the beginning of the class to ensure student accountability and completion of the out-of-class activities. Instead, faculty can encourage interactions between peers and instructors using the clickers or IRAT/GRAT scores for in-class assessment. Summative and formative assessments of students’ knowledge and skills (e.g., problem-based learning and solving complex case scenarios) are necessary to move to the next stage of learning. Hanna and Dettmer [81, 82] suggested that faculty should strive to develop a range of assessment strategies that match all aspects of their instructional plans. Moreover, instead of trying to differentiate between formative and summative assessments, FC instructors may find greater benefit in planning their assessment strategies to match the instructional goals and objectives at the beginning of the semester.

## Evaluation of the Flipped Classroom Model

The assessment of student performance following FC instruction is an evolving area of research. A review of the existing literature on using FC in medical education showed that FC has a significant impact on students’ attitudes and performance. Table 3 summarizes the effects of FC implementation on students’ perceptions and performance in medical education.

**Table 3:** Implementation of the flipped classroom (FC) and its impact on student performance in medical education

Study	Discipline and experimental design and outcome variables	Study conclusions or performance outcomes	
		Students’ perceptions	Students’ performance
1. Rui et al. (2017)	ECG learning in medical diagnostics used randomized controlled trial to compare FC to lecture-based learning. Students’ examination scores and self-administered questionnaire were analyzed	Students had positive attitudes toward FC	Students in the FC group scored significantly higher (*t* = 4.549, *p* = 0.0000)
2. Street et al. (2015)	Physiology learning in preclerkship years used quasi-experimental design to compare students’ examination performance and course evaluation ratings across cohorts	Student satisfaction with the course improved significantly (*d* = 0.304, *p* = 0.005)	Only slight improvement in student performance (*p* = 0.026)
3. Liebert et al. (2016)	Investigates medical students’ perception of simulation-based FC for the surgery clerkship. Students filled in end-of-clerkship survey regarding their perception of the curriculum. Quantitative analysis of the Likert responses and qualitative analysis of narrative responses performed	Students’ perceptions of the simulation-based FC for surgery clerkship were positive, with 90% rating it excellent	N/A
4. Morton et al. (2017)	Determined if the FC relative to LC increases student performance on the anatomy items categorized by Bloom’s taxonomy were compared between students using FC and LBL	N/A	FC students performed better on anatomy items testing on analysis category than LC students (*U* = 4243.00, *p* = 0.030, *r* = 0.19). For anatomy items testing categories of knowledge and apply, the difference between LC and FC was not significant
5. O’Connor et al. (2016)	Radiology clerkships compare effects of FC to didactic instruction on students’ academic achievement, task value, and achievement emotions. The study used linear mixed method effect analysis to examine relationship between test scores and instruction type	FC improved academic achievement, task value, and achievement emotions (all *p* < 0.01) compared with lecture-based learning	No baseline difference in the test scores of FC and LC students. The mean post-test minus pre-test scores were higher in FC compared to LC (10.5% higher, *p* = 0.013)
6. Morgan et al. (2015)	Obstetrics and gynecology clerkship (MS3 students watched 4 short videos prior to class time active learning session). Student satisfaction on 2 aspects was obtained — lecture assessment on GynecOnco topics and aggregate student performance on subject questions on NBME exams compared before and after FC implementation	Student satisfaction for FC instruction with pre-class videos (3.31 ± 0.65) and class-time cases (3.61 ± 0.62) for obstetrics and gynecology clerkship was positive	No significant difference in performance on gynecological oncology questions of NBME examination compared with national performance (8.3 versus 8.4%, *p* = 0.99)
7. Belfi et al. (2015)	Radiology clerkships (MS3) used radiology modules to an open-source website. Students exposed to different teaching methods blended learning, traditional lectures, and independent learning. Students completed pre-course and post-course knowledge assessments and surveys	Student perceptions of the online modules and the self-paced interactivity were very positive	Blended learning achieved greater pre-test-to-post-test scores (*p* = 0.0060) compared with lecture-based learning
8. Fleagle et al. (2018)	In dissection-based dental anatomy, lab students used online material to prepare for the anatomy lab and in-class time used for integrative group activity. Scores on lab examination were compared for the 3 years before the current change and 3 years after	Students expressed a high degree of satisfaction even though FC created more homework prior to laboratories	Final anatomy lab scores were significantly higher in the post-flipped classes (*p* = 0.04)
9. Cheng et al. (2017)	Histology curriculum in Chinese medical program compared students using FC format and traditional lectures for basic tissue section of the course and organ system section of the course. The academic performance of 2 groups was compared and questionnaire administrated to FC group	Majority of students enjoyed FC learning experience (4.37 ± 0.68)	In the flipped classroom format group, the percent of correct scores for the organ systems section was significantly higher (*p* = 0.037, Cohen’s *d* = 0.666) than for the basic tissues section. In comparison, for the traditional classroom student group, the percentage of correct scores was not statistically different for the organ systems versus basic tissues sections

Several studies show that FCs significantly improved student performance [17, 19, 83–87]. In one study, which compared student performance on an anatomy examination containing 28 multiple-choice questions, the class of students who learned the material using the FC format performed better than those who learned the material via lecture-based learning [17, 26]. Fleagle et al. [26] examined FC pedagogy among dental gross anatomy students who prepared for their out-of-class dissection laboratories by watching 3D anatomy videos, abbreviated dissection instructions, dissection videos, and atlas figures. The study compared the students’ scores on laboratory examinations for the 3 years before and after the FC curriculum and found that the final semi-cumulative laboratory examination scores were significantly higher in the post-FC classes than in the pre-FC classes. A radiology clerkship study that used FC pedagogy asked third-year medical students to watch e-learning modules and complete pre- and post-course knowledge assessments. The study found that students in FC courses achieved higher pre-test-to-post-test performance compared with students in lectures alone [86]. Furthermore, students have shared positive perceptions of their FC experiences in several disciplines: e.g., ECG learning in medical diagnostics [85], physiology [83], surgery [68], anatomy [17, 26, 87], histology [19], radiology [86, 88], and obstetrics and gynecology [84].

One study used a self-administered questionnaire to evaluate the students’ attitudes, total learning time, and conditions under the FC teaching method [85]. The results showed that 72% of the respondents thought that the FC was an effective teaching model worthy of promotion in the future. Street et al. [83] studied a medical physiology course and compared the students’ FC experience with their lecture-based learning experience. The study found that the FC increased students’ analytical thinking by 58.5% and problem-solving by 61.5% compared with the traditional classroom. An interinstitutional study by O’Connor [88] compared didactic and FC radiology sessions and found that 33% of students in the didactic sessions and 60% of students in the FC sessions strongly agreed with the statement that “I understand the material taught in these sessions.” Morgan et al. [84] also found highly positive student satisfaction ratings for FC pre-class and in-class activities in an obstetrics and gynecology clerkship: the student satisfaction ratings on a 4-point Likert scale to the survey question, “Overall I would rate this activity as positive,” were 3.44 ± 0.63 for the pre-class activities and 3.65 ± 0.54 for the in-class activities.

## Limitations

Several limitations of FC should be considered. First, the self-directed pre-class work requires better methods of data collection regards students’ completion of tasks. Example, did students get access to the videos? Did students watch the videos till completion? Therefore, many times it remains unknown if the videos influenced the study or were there any external factors that supported student learning of understanding the material. Secondly, majority of FC studies are single institutional studies; in future, large multi-institutional randomized studies and more precise tracking methods to track various data sets are needed. Thirdly, longitudinal studies on the impact of FC on long-term knowledge retention including the impact of student performance on licensure exams USMLE and NBME shelf-exams are essential to bring FC as a mainstream instruction method. Furthermore, it will be interesting to gain insights on the relationship of FC instruction and lifelong learning.

## Conclusions

The FC boosts students’ self-reported confidence in the material and improves their performance, thus representing an important and exciting advancement in medical education. This review highlights several rigorous and relevant publications on FC theories and applications to serve as both a resource and summary for educators. It also provides insight into learners’ experiences with the FC model in medical education and highlights several elements that may be important for the effective design and implementation of this model. FC instructional methods demonstrated a modest increase in improvement in learning in disciplines like radiology, physiology, and anatomy. The existing studies on improvement in learning can serve as a baseline framework to develop additional research on FC in other disciplines of medicine. Student feedback on their FC experiences would help optimize the learning outcomes, ensuring that this pedagogical method remains flexible, transferable, and relevant. Future research on cost-effectiveness, non-cognitive skill development (e.g., teamwork, empathy, communication, adaptability), learning space design, the long-term impact on knowledge retention, and faculty development will advance the impact of the FC.
